# Doubly Covariance Matrix Reconstruction Based Blind Beamforming for Coherent Signals

**DOI:** 10.3390/s20123595

**Published:** 2020-06-25

**Authors:** Zhuang Xie, Chongyi Fan, Jiahua Zhu, Xiaotao Huang

**Affiliations:** 1 College of Electronic Science and Technology, National University of Defense Technology, Changsha 410073, China; xiezhuang18@nudt.edu.cn (Z.X.); xthuang@nudt.edu.cn (X.H.); 2 College of Meteorology and Oceanography, National University of Defense Technology, Changsha 410073, China; zhujiahua1019@hotmail.com

**Keywords:** multipath arrivals, blind beamforming, interference-plus-noise covariance matrix, composite steering vector

## Abstract

This paper proposes a beamforming method in the presence of coherent multipath arrivals at the array. The proposed method avoids the prior knowledge or estimation of the directions of arrival (DOAs) of the direct path signal and the multipath signals. The interferences are divided into two groups based on their powers and the interference-plus-noise covariance matrix (INCM) is reconstructed through the doubly covariance matrix reconstruction concept. The composite steering vector (CSV) that accounts for the direct path signal and multipath signals is estimated as the principal eigenvector of the sample covariance matrix with interferences and noise removed. The optimal weight vector is finally computed using the INCM and the CSV. The proposed method involves no spatial smoothing and avoids reduction in the degree of freedom. Simulation results demonstrate the improved performance of the proposed method.

## 1. Introduction

Adaptive beamforming is a well concerned research topic of array signal processing, with wide spread applications in radar, sonar signal processing and other fields [1,2,3,4,5,6]. Traditional beamforming mainly depends on the prior information about the array manifold and desired signals to form a sharper beam for the suppression of interferences and noise [7,8].

Conventional adaptive beamforming techniques are effective under the assumption of the independence among the received signals. However, in real scenarios, the desired signal may be received as a group of coherent signals from multiple directions. For instance, in communication systems, due to the multipath effect, the desired signal may be overlapped by the reflected/scattered interferences from, say, ground or sea surface, which leads to the coherence of the received signals. In this case, the conventional adaptive beamformer may suppress the desired signal and degrade the performance of the beamformer. A class of pre-processing methods for reducing the influence of signal correlation are proposed. The well-known spatial smoothing method divides array sensors into subarrays and averages data covariance matrices of subarrays to decrease the coherence of signals [9]. There are also techniques like temporal smoothing [10] and subcarrier smoothing [11], etc. The main concept of these methods is to decorrelate the coherence and make processed data available for conventional beamforming.

In practical applications, the prior information about the desired signal is usually unavailable and the performance of the traditional approach may be significantly deteriorated. The blind beamforming technique can maintain satisfactory performance when the parameters are not precisely known, which dramatically improves the robustness and output performance of the beamforming algorithm.

Blind identification was first applied on beamforming by using blind identification to estimate the steering vector without the prior knowledge of the array manifold [12]. Based on the array structure, a blind beamforming method was proposed using estimating signal parameters via rotational invariance techniques (ESPRIT) to calculate the weighting matrix for each signal [13]. This method is based on an algebraic structure and has an analytical solution formula, which leads to small calculation complexity. In the absence of array errors, the performance of this method is close to that of ideal minimum variance distortionless response (MVDR) beamformer. However, once array distortions such as channel amplitude and phase errors are taken into account, the output performance is severely deteriorated. In addition, because the rotation-invariant technique is used, this method is only suitable for uniform linear arrays. Constant modulus algorithm (CMA) was first proposed for blind equalization and extended to blind beamforming. The constant modulus algorithm uses the constant modulus of the desired signal without training data to construct a constant modulus cost function for the received signal. The stochastic gradient descent CMA (SGD-CMA) [14] and recursive least squares CMA (RLS-CMA) [15] are two typical representatives of the constant modulus algorithms, but they can only be used for a single CM signal. Another type of constant modulus algorithm is the analytic constant modulus algorithm (ACMA) [16,17,18]. The basic idea is to use the constant modulus of the signal to directly perform algebraic operation on the data matrix of the received signal to obtain a spatial filter. This algorithm can obtain the weighting vectors of multiple spatial filters simultaneously, while the number of array elements should be no less than the square of the number of sources, and it is not effective for non-circular signals. There are also some novel machine learning approaches for blind beamforming, which can be effective in specific scenarios [19,20,21].

Wang et al. [22] proposed a decorrelation-based blind (DBB) beamformer to achieve blind beamforming when the desired signal is received by the array as coherent signals. The desired signal is assumed to arrive the array at multiple directions and the signals in these directions are coherent with each other. The DBB algorithm in fact performs blind beamforming based on the data after spatial smoothing process, whose performance is closely related to the spatial smoothing algorithm. Zhang et al. [23] estimated the composite steering vector (CSV) of multipath coherence signals via the eigendecomposition of the echo data covariance matrix, which is called eigendecomposition-based blind (EBB) beamformer. However, the EBB beamformer requires that each interference should be stronger enough than the desired signal, otherwise the performance deteriorates.

In this paper, we propose the doubly covariance matrix reconstruction concept to achieve blind beamforming for coherent signals. The main problem is to reconstruct the interference-noise covariance matrix (INCM), and obtain the CSV of desired coherent signals, and then the weighting vector of MVDR beamformer can be calculated to eliminate interferences and noise which retains the desired signal sequence. Specifically, our approach consist of the following steps: First, the covariance matrix of comparable-to-desired-signals-in-power (CDSP) interferences is calculated after estimating their power and directions of arrivals (DOAs) through the spatial spectrum, then the covariance matrix of normal interferences is reconstructed using eigenvectors of a transformed form of sample covariance matrix (SCM). Second, with the INCM obtained by summing the covariance matrices of interference and noise, the CSV is estimated as a unblinding process, that is, through the eigenvalue decompostition of SCM with interference and noise components removed. Finally the weighting vector is determined by the INCM and CSV. Importantly, for the array, the degree of freedom is preserved since no smooth process is involved in the proposed approach. Simulation results demonstrate that output signal-to-interference-plus-noise ratio (SINR) of proposed approach attains satisfied performance in terms of blind beamforming for coherent signals and outperforms the compared approaches.

The rest of this paper is organized as follows: In Section 2, some indispensable formulas about the model of receiver sensors array are given and necessary background about beamformer for coherent signals is presented. In Section 3, a new blind beamforming approach for coherent signals is put forward in detail. In Section 4, the output SINR performance of the proposed approach is evaluated through simulations and compared with those of some existing approaches. Section 5 gives the conclusion of this paper and expectation of future work. Throughout this paper, E[·] is the statistical expectation operator and ${\left(\cdot \right)}^{H}$ stands for the conjugate transpose operator. ${\left(\cdot \right)}^{T}$ denotes the transpose operator and I is identity matrix.

## 2. Problem Formulation

Consider a uniform linear array (ULA) consists of *M* radar receiving elements. Assume there are Q+1 narrowband signal sources in the far-field uncorrelated with each other, including one desired source and *Q* interference sources. In practice, due to multipath propagation, the desired signal arrives at the array from *P* different directions, and the DOAs of the direct path signal and multipath signals are $\theta_{1},\theta_{2},\ldots ,\theta_{P}$. The DOAs of the *Q* interference signals are $\phi_{1},\phi_{2},\ldots ,\phi_{Q}$. Then at the *k*th snapshot, the data vector received by the array can be expressed as

(1) x(k)=AS(k)+n(k)

where $S\left(k\right)={\left[\beta \cdot s\left(k\right),I\left(k\right)\right]}^{T}$ denotes the echo signal matrix at instant *k*, and s(k) represents the original signal sequence radiated by the desired source with power $\sigma_{s}^{2}$. $\beta =[\beta_{1},\beta_{2},\ldots ,\beta_{P}]$ is the phase and amplitude gain vector with element $\beta_{p}$ being the phase and amplitude gain during the propagation in the *p*th way. $I\left(k\right)=[I_{1}\left(k\right),I_{2}\left(k\right),\ldots ,I_{Q}\left(k\right)]$ is the interference vector at instant *k* and the power of *q*th interference signal is $\sigma_{Iq}^{2}$. $A=[A_{s},A_{I}]$ stands for the steering matrix which contains the steering vector for each DOA. $A_{s}=\left[a\left(\theta_{1}\right),a\left(\theta_{2}\right),\ldots ,a\left(\theta_{P}\right)\right]$ is the steering matrix of desired signal corresponding to *P* propagation paths and $A_{I}=\left[a\left(\phi_{1}\right),a\left(\phi_{2}\right),\ldots ,a\left(\phi_{Q}\right)\right]$ is the steering matrix of *Q* interferences, respectively. n(k) is a zero mean, uncorrelated additive white Gaussian noise with variance $\sigma_{n}^{2}$. In this paper, the noise in each channel is considered to be uncorrelated with the desired signal and interferences.

With an infinite number of snapshots, the theoretical covariance matrix of data received by the array can be expressed as

(2) $$\begin{array}{cc} R_{x}= & E[x\left(k\right)x^{H}\left(k\right)]\\ = & AE\left[S\left(k\right)S^{H}\left(k\right)\right]A^{H}+E\left[n\left(k\right)n^{H}\left(k\right)\right]\\ = & \sigma_{s}^{2}A_{s}\beta^{H}\beta A_{s}^{H}+A_{I}E\left[I^{H}\left(k\right)I\left(k\right)\right]A_{I}^{H}+\sigma_{n}^{2}I\\ = & \sigma_{s}^{2}VV^{H}+A_{I}\Sigma_{I}A_{I}^{H}+\sigma_{n}^{2}I\\ = & R_{s}+R_{I}+R_{n} \end{array}$$

where $V=A_{s}\beta^{H}$ is CSV of the coherent signals. $\Sigma_{I}=\mathrm{diag}\left[\sigma_{I1}^{2},\ldots ,\sigma_{IQ}^{2}\right]$ is a diagonal matrix with diagonal elements $\sigma_{Iq}^{2}=E\left[I_{q}^{H}\left(k\right)I_{q}\left(k\right)\right]$.

The beamforming technique can be applied to eliminate the interference and noise components in the received data while keep the desired signal unaffected through the weighted summation of data of different channels. The output of beamformer with interference and noise suppressed at instant *k* can be written as

(3) $$y\left(k\right)=w^{H}x\left(k\right)$$

where w is the M×1 weighting vector.

The constraints of the well-known MVDR beamformer are given as

(4) $$\min w^{H}R_{I+n}w\;s.t.w^{H}V=1$$

where $R_{I+n}=R_{I}+R_{n}$ is the INCM. The solution weighting vector of MVDR beamformer is

(5) $$w=\frac{R_{I+n}^{-1}V}{V^{H}R_{I+n}^{-1}V}$$

In practice, the INC matrix $R_{I+n}$ is usually unavailable, and it is replaced with the SCM

(6) $${\hat{R}}_{x}=\frac{1}{K}\sum_{k=1}^{K}x\left(k\right)x^{H}\left(k\right)$$

where *K* is the number of snapshots and x(k) is the data vector of *k*th snapshot. The beamformer above now turns into minimum power distortionless response (MPDR) beamformer, which is also called sample matrix inversion (SMI) beamformer. It is known that when desired signal is present in the covariance matrix, the performance of beamformer deteriorates.

## 3. Proposed Approach

In this part, the proposed blind beamforming approach is introduced. From Section 2, we can conclude that the estimation of CSV V and INCM $R_{I+n}$ are the main problems of blind beamforming in presence of coherent signals. In the following content, detailed illustration about the corresponding estimation process in the proposed approach is given.

In real application, the interferences we usually consider as “normal interferences” are those who obscure the desired signal due to their much larger power. On the other hand, there are also another kind of interferences (we named them as “CDSP interferences” in the later) with power comparable to that of the desired signal, that may also be regarded as false signals and influence the performance of beamforming. Therefore, we further extend the signal model in (1) to a more generalized situation, and give the following array signal model for better illustration. Assuming that *Q* interferences are composed of $Q_{1}$ CDSP interferences (i.e., for $q=1,\ldots ,Q_{1}$, $\sigma_{Iq}^{2}$ is comparable to the desired signal power $\sigma_{s}^{2}$) and $Q-Q_{1}$ normal interferences (i.e., for $q=Q_{1}+1,\ldots ,Q$, $\sigma_{Iq}^{2}\gg \sigma_{s}^{2}$), then (2) can be rewritten as

(7) $$\begin{array}{cc} R_{x}= & R_{s}+A_{I1}\Sigma_{I1}A_{I1}^{H}+A_{I2}\Sigma_{I2}A_{I2}^{H}+R_{n}\\ = & R_{s}+R_{I1}+R_{I2}+R_{n} \end{array}$$

where $\Sigma_{I1}=diag\left[\sigma_{I1}^{2},\ldots ,\sigma_{IQ_{1}}^{2}\right]$, $\Sigma_{I2}=diag\left[\sigma_{IQ_{1}+1}^{2},\ldots ,\sigma_{IQ}^{2}\right]$, $A_{I1}$ and $A_{I2}$ are steering matrices of $Q_{1}$ CDSP interferences and $Q-Q_{1}$ normal interferences, respectively.

### 3.1. Doubly Covariance Matrix Reconstruction

#### 3.1.1. Covariance Matrix Reconstruction of CDSP Interferences

We estimate the noise power $\sigma_{n}^{2}$ as the minimum eigenvalue of ${\hat{R}}_{x}$ [24]. Since the noise in the paper is assumed to be white Gaussian, the noise covariance matrix can be reconstructed as

(8) $$\begin{array}{c} {\hat{R}}_{n}={\hat{\sigma }}_{n}^{2}I \end{array}$$

Capon spatial spectrum can be utilized to give the spatial spectrum distribution over the whole space, where the interference DOAs can be estimated from the location of the peaks [25,26]. Moreover, the power estimation of the interferences can be operated from the value of the peaks. The Capon spectrum is applied to reconstruct the covariance matrix for the CDSP interferences, the basic expression of Capon spectrum is given as

(9) $$\begin{array}{c} P\left(\phi \right)=\frac{1}{a^{H}\left(\phi \right){\hat{R}}_{x}^{-1}a\left(\phi \right)} \end{array}$$

The basis of the spatial spectrum is the ordinary steering vector a(ϕ), so the peak of desired signal is not in the spread spectrum. By using Capon spectrum to search in the whole spectrum, we obtain a series of peaks. Usually, the number of these peaks is larger than the truth number of interferences *Q* because there are some peaks formed by noise. Since the peaks formed by noise are far lower than the peaks corresponding to real interferences in the spectrum [27], it is applicable to set a threshold to choose the peaks of real interferences and determine the number of interferences *Q*. Considering the fact that there are *M* channels in the receiver array, the value of threshold can be set as ${\hat{\sigma }}_{n}^{2}/M$. If the value of the peak is lower than ${\hat{\sigma }}_{n}^{2}/M$, it is considered as a non-interfering peak and removed.

After the selection, the remaining peaks contain both normal and CDSP interferences. The small value peaks are chosen out as peaks of CDSP interferences, i.e., the peaks of which the values are relatively larger are considered belonging to the normal interferences. Actually there is no precise limit to differ the relatively larger and relatively small peaks. Usually, we can give an experiential value of this limit through experimental measurements, which has been further explored in the Example 1 of simulation part. The number of CDSP interferences $Q_{1}$ is obtained as the number of relatively small values peaks. The values of the $Q_{1}$ selected peaks are considered as the power estimates of the CDSP interferences, and the corresponding angles ${\hat{\phi }}_{1},\ldots ,{\hat{\phi }}_{Q_{1}}$ can be regarded as their DOAs. The covariance matrix of $Q_{1}$ CDSP interferences can be reconstructed as

(10) $$\begin{array}{c} {\hat{R}}_{I1}=\sum_{q=1}^{Q_{1}}\frac{a\left({\hat{\phi }}_{q}\right)a^{H}\left({\hat{\phi }}_{q}\right)}{a^{H}\left({\hat{\phi }}_{q}\right){\hat{R}}_{x}^{-1}a\left({\hat{\phi }}_{q}\right)} \end{array}$$

#### 3.1.2. Covariance Matrix Reconstruction of Normal Interferences

Before we proceed to the following content about the reconstruction of $R_{I2}$, some necessary basis of our approach should be illustrated. The matrix theorem about rank-one modification of the symmetric eigenproblem is used in [23], we now extend it to a more generalized situation. First, the content of original theorem should be introduced.

**Theorem** **1.** *Suppose B is a known Hermitian M×M matrix, b is a M×1 complex vector. $\hat{B}=B+pbb^{H}$, where p∈R. Then the kth eigenvalue of $\hat{B}$ can be given as*

(11) $${\hat{\lambda }}_{k}=\lambda_{k}+p\mu_{k}$$

*where $\lambda_{k}$ is the kth eigenvalue of B, $\sum_{k=1}^{M}\mu_{k}=1$ and $0\leq \mu_{k}\leq 1$. Denote the eigenvalue decomposition of B as $B=QDQ^{H}$, then the corresponding kth eigenvector of $\hat{B}$ is*

(12) $$\hat{q_{k}}=Q\frac{D_{k}^{-1}z}{∥D_{k}^{-1}{z∥}_{2}}$$

*where $D_{k}=D-{\hat{\lambda }}_{k}I$, $z=Q^{H}b$. Proof of Theorem 1 can be referred to [28].*

According to the above theorem, if $\lambda_{k}\gg p$, the change of the eigenvalue of B can be ignored after adding such a rank-one matrix $pbb^{H}$. On the other hand, it is obvious that $\hat{q_{k}}$ is a linear combination of all eigenvectors of B, (i.e., $q_{1},q_{2},\ldots ,q_{M}$). The *m*th diagonal entry of $D_{k}^{-1}$ is ${\left(\lambda_{k}-{\hat{\lambda }}_{k}\right)}^{-1}$, when $\lambda_{k}$ is sufficiently large ${\left[D_{k}^{-1}\right]}_{k,k}$ would be the largest entry, thus $\hat{q_{k}}\approx q_{k}$ can be derived since $q_{k}$ is the dominant contributor to $\hat{q_{k}}$.

This theorem can be extended to a generalized situation. For a Hermitian matrix B, its dominant eigenvalues and corresponding eigenvectors would not be significantly changed by adding *n* rank-one matrices $p_{1}b_{1}b_{1}^{H},p_{2}b_{2}b_{2}^{H},\ldots ,p_{n}b_{n}b_{n}^{H}$, where $p_{1},p_{2},\ldots ,p_{n}\in R$ are small enough, $b_{1},b_{2},\ldots ,b_{n}\in R^{M}$. The above conclusion is obvious and can be proved as follows.

Since rank-one matrices $p_{1}b_{1}b_{1}^{H},p_{2}b_{2}b_{2}^{H},\ldots ,p_{n}b_{n}b_{n}^{H}$ are all Hermitian matrices, $B+\sum_{i=1}^{i=n-1}p_{i}b_{i}b_{i}^{H}$ is a Hermitian matrix and its dominant eigenvalues and corresponding eigenvectors would not be significantly affected by adding rank-one matrix $p_{n}b_{n}b_{n}^{H}$. Similarly, for $B+\sum_{i=1}^{i=n-2}p_{i}b_{i}b_{i}^{H}$, the dominant eigenvalues and corresponding eigenvectors approximate that of $B+\sum_{i=1}^{i=n-1}p_{i}b_{i}b_{i}^{H}$. As a corollary, these large eigenvalues and corresponding eigenvectors of B stay approximately unchanged after adding these rank-one matrices $p_{1}b_{1}b_{1}^{H},p_{2}b_{2}b_{2}^{H},\ldots ,p_{n}b_{n}b_{n}^{H}$.

Suppose the eigenvalue decomposition of $R_{I2}$ is given as

(13) $$R_{I2}=U_{I2}\Lambda_{I2}U_{I2}^{H}$$

where $U_{I2}=\left[u_{I1},u_{I2},\ldots ,u_{IM}\right]$ is the eigenvector matrix, $\Lambda_{I2}=diag\left[\lambda_{I1},\lambda_{I2},\ldots ,\lambda_{IM}\right]$ is the eigenvalue matrix. These eigenvalues are arranged in descending order, (i.e., $\lambda_{I1}\geq \lambda_{I2}\geq \ldots \lambda_{IQ-Q_{1}}\gg \lambda_{IQ-Q_{1}+1}=\lambda_{IQ-Q_{1}+2}=\cdots =\lambda_{IM}=0$).

Assume the eigenvalue decomposition of ${\tilde{R}}_{x}=R_{s}+R_{I1}+R_{I2}$ is given as

(14) $$\begin{array}{c} {\tilde{R}}_{x}=R_{x}-\sigma_{n}^{2}I=U\Lambda U^{H} \end{array}$$

where $U=[u_{1},u_{2},\ldots ,u_{M}]$, $\Lambda =diag[\lambda_{1},\lambda_{2},\ldots ,\lambda_{M}]$.

According to the above theorem, if $\sigma_{Iq}^{2}\gg \sigma_{s}^{2}$, for $q=Q_{1}+1,\ldots ,Q$, then the $Q-Q_{1}$ dominant eigenvalues and the corresponding eigenvectors of $R_{I2}$ would not be significantly affected by adding $R_{s}+R_{I1}$. Therefore, $R_{I2}$ can be approximated by the dominating $Q-Q_{1}$ large eigenvalues and the corresponding eigenvectors of ${\tilde{R}}_{x}$. Defining ${\hat{\tilde{R}}}_{x}={\hat{R}}_{x}-{\hat{\sigma }}_{n}^{2}I$ as the estimate of ${\tilde{R}}_{x}$, where ${\hat{\sigma }}_{n}^{2}$ is the estimate of noise variance, we derive the following approximate equation

(15) $$\begin{array}{c} {\hat{R}}_{I2}={\hat{U}}_{I2}{\hat{\Lambda }}_{I2}{\hat{U}}_{I2}^{H} \end{array}$$

where ${\hat{\Lambda }}_{I2}$ is the eigenvalue matrix containing the $Q-Q_{1}$ largest eigenvalues of ${\hat{\tilde{R}}}_{x}$ and ${\hat{U}}_{I2}$ includes $Q-Q_{1}$ corresponding dominant eigenvectors.

It should be noted that during the reconstruction of the $Q-Q_{1}$ normal interferences, there is an prerequisite that the $Q-Q_{1}$ normal interferences are strong enough in power. However, in the Example 1 of the simulation part, the performance of proposed approach is proved to attain optimal when the normal interferences are 10 dB stronger than the desired signals. Situations with this power difference are common in real scene. The more complex scenarios are out of the scope of this paper and are listed as a further extension work.

### 3.2. Unblinding Desired Coherent Signals

Based on the reconstructed interference covariance matrices ${\hat{R}}_{I1}$ and ${\hat{R}}_{I2}$, the INCM can be estimated as

(16) $${\hat{R}}_{I+n}={\hat{R}}_{I1}+{\hat{R}}_{I2}+{\hat{\sigma }}_{n}^{2}I$$

Therefore, the estimation of desired signal covariance matrix $R_{s}$ can be obtained as

(17) $$\begin{array}{c} {\hat{R}}_{s}={\hat{R}}_{x}-{\hat{R}}_{I+n} \end{array}$$

It is known that multiplying V by a nonzero scalar would not affect the performance of beamformer. Therefore, the CSV of desired coherent signals V can be estimated as the eigenvector of ${\hat{R}}_{s}$ corresponding to the largest eigenvalue as

(18) $$\begin{array}{c} \hat{V}=P\left[{\hat{R}}_{s}\right] \end{array}$$

where the operator P[·] denotes the eigenvector of the matrix corresponding to the principal eigenvalue.

The weighting vector of the proposed blind beamforming approach can thereby be obtained and given as

(19) $$\begin{array}{c} w=\frac{{\hat{R}}_{I+n}^{-1}\hat{V}}{{\hat{V}}^{H}{\hat{R}}_{I+n}^{-1}\hat{V}} \end{array}$$

The detailed procedures of the proposed blind beamforming approach are summarized in Algorithm 1 shown as follows.
**Algorithm 1** Steps of doubly covariance matrix based blind beamforming approach.**Part 1:** doubly covariance matrix reconstruction ***1.*** eigendecomposing the SCM ${\hat{R}}_{x}$. ***2.***
${\hat{\sigma }}_{n}^{2}$ = minimum eigenvalue of ${\hat{R}}_{x}$. ***3.***
*Q* = number of spectrum peaks sufficiently larger than ${\hat{\sigma }}_{n}^{2}/M$. ***4.***
$Q-Q_{1}$ = spectrum peaks of dominant value among *Q* peaks. ***5.*** reconstructing covariance matrix for the $Q_{1}$ CDSP interferences as ${\hat{R}}_{I1}=\sum_{q=1}^{Q_{1}}\frac{a\left({\hat{\phi }}_{q}\right)a^{H}\left({\hat{\phi }}_{q}\right)}{a^{H}\left({\hat{\phi }}_{q}\right){\hat{R}}_{x}^{-1}a\left({\hat{\phi }}_{q}\right)}$ ***6.*** decomposing SCM with noise components removed as ${\hat{\tilde{R}}}_{x}=\hat{U}\hat{\Lambda }{\hat{U}}^{H}$ ***7.*** reconstructing covariance matrix for the $Q-Q_{1}$ normal interferences as ${\hat{R}}_{I2}={\hat{U}}_{I2}{\hat{\Lambda }}_{I2}{\hat{U}}_{I2}^{H}$.**Part 2:** unblinding desired coherent signals. ***8.*** reconstructing the INC matrix ${\hat{R}}_{I+n}$ according to (16) and obtaining ${\hat{R}}_{s}$ based on (17). ***9.*** estimating the CSV of desired coherent signals $\hat{V}=P\left[{\hat{R}}_{s}\right]$. **Final** calculating the weighting vector $w=\frac{{\hat{R}}_{I+n}^{-1}\hat{V}}{{\hat{V}}^{H}{\hat{R}}_{I+n}^{-1}\hat{V}}$

## 4. Simulation

In this section, simulations are provided to examine the performance of proposed approach via several examples. A ULA of 35 sensors is considered in the simulation for all examples, the covariance of the white Gaussian noise is assumed to be $\sigma_{n}^{2}=I$. The distance between the adjacent elements is set as half of the wavelength. Suppose that the desired signal reaches the array in form of a group of coherent signals from multiple directions. Here, we consider the situation that the desired signals propagate through three different paths with phase and amplitude gain $\beta_{1}=0.8j$, $\beta_{2}=0.4j$, $\beta_{3}=-0.2j$. Their DOAs are $\theta_{1}=-30^{\circ }$, $\theta_{2}=-40^{\circ }$, $\theta_{3}=-50^{\circ }$ respectively.

To better demonstrate the performance of the proposed approach, three representative blind beamformers—the EBB approach in [23], the MVDR-Smooth approach in [27], and the optimal beamformer are compared with the proposed approach in the following simulations. For the MVDR-Smooth approach, 10 subarrays are applied for smoothing. Moreover, it should be noted that the optimal beamformer is the MVDR beamformer with known CSV. In all simulation results, 300 Monte-Carlo trials are run for every single point.

### 4.1. Simulation Example 1

As stated before, the normal interferences should be sufficiently stronger than the desired signal so that the proposed covariance matrix reconstruction method for the normal interferences can be applied. In the first example, simulation is performed to examine how large the interference-to-noise ratio (INR) should be to meet the restriction.

**Figure 1:**
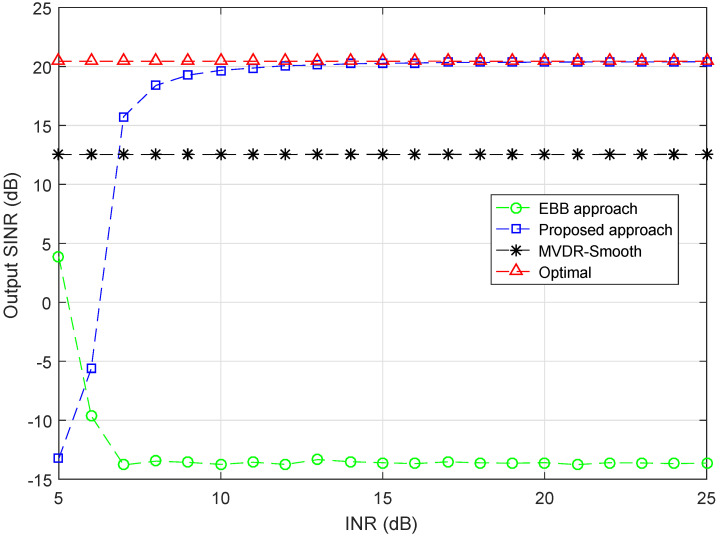
Output SINR versus INR (Group 2), INR (Group 1) = 5 dB, SNR = 0 dB.

Q=6 interferences are considered in this example and are divided into two groups for illustration convenience, i.e., Group 1: $I_{1},I_{2},I_{3}$ with DOAs $\left\{20^{\circ },30^{\circ },40^{\circ }\right\}$ and Group 2: $I_{4},I_{5},I_{6}$ with DOAs $\left\{50^{\circ },60^{\circ },70^{\circ }\right\}$. In this example, the INR of Group 1 is fixed at 5 dB. With signal-to-noise ratio (SNR) fixed at 0 dB, the output SINR curves versus INR of Group 2 varying from 5 dB to 25 dB are plotted in Figure 1. As is shown in the figure, when the INR is low, the performance of proposed approach deteriorates, the output SINR is far away from optimal as expected. This is because at low INRs, the approximation ${\hat{R}}_{I2}={\hat{U}}_{I2}{\hat{\Lambda }}_{I2}{\hat{U}}_{I2}^{H}$ no longer holds and the estimation of INC matrix will be inaccurate. However, proposed approach outperforms the compared approaches when INR > 7 dB and attains the optimal when INR > 10 dB. This proves the restriction for real application of the proposed approach is not hard to meet since the interferences are stronger than 10 dB in many scenarios. On the other hand, the EBB beamformer cannot perform well when there are interferences comparable to desired signals in power, even the INR is high, the output SINR of MVDR-smooth approach maintains stable at approximately 13 dB at all INRs.

### 4.2. Simulation Example 2

**Figure 2:**
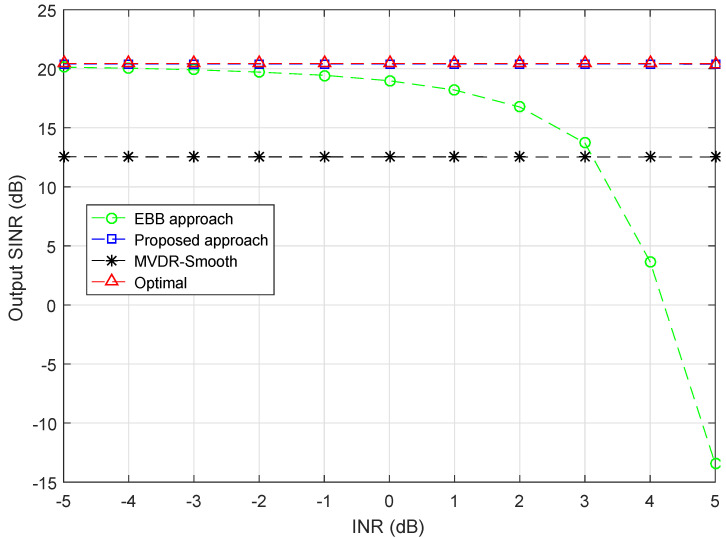
Output SINR versus INR (Group 1), INR (Group 2) = 25 dB, SNR = 0 dB.

To further investigate the performance of proposed approach, fixing the INR of Group 2 as 25 dB while keeping other conditions unchanged, in Figure 2, we plot the output SINR curves versus INR of Group 1 varying from −5 dB to 5 dB. As can be seen from the figure, the proposed approach stays close to the optimal SINR over a large range from −5 dB to 5 dB. For EBB approach, it performs well and is close to the optimal when INR is low. However, it can be observed that the existing of CDSP interferences degrades its performance, and is more significant as the INR gets larger. When INR >3 dB, it is inferior to the MVDR-Smooth approach. When INR =5 dB, the output SINR of EBB approach is −15 dB, which is far away from the optimal 20.5 dB. On the other hand, the performance of MVDR-Smooth approach is more stable, it is insensitive to the variation of INR and maintains an output SINR of 12.5 dB. It can be seen from Figure 2 that proposed approach performs well in this example and displays the best performance among all the approaches, it stays close to the optimal at all INRs.

### 4.3. Simulation Example 3

**Figure 3:**
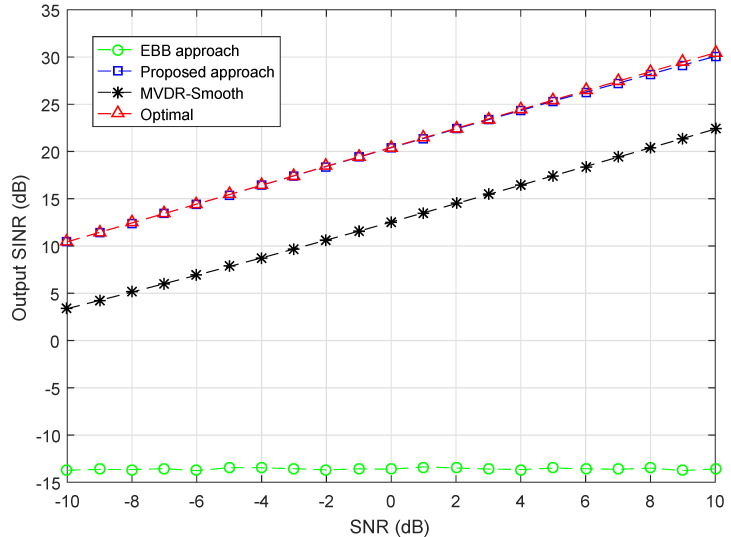
Output SINR versus SNR, ISR (Group 1) = 5 dB, ISR (Group 2) = 25 dB.

In the third example, with the interference-to-signal ratio (ISR) of two interference groups fixed and other conditions unchanged, we explore the performance of proposed approach at various SNRs. ISRs are set as 5 dB and 25 dB for Group 1 and Group 2, respectively. The SNR is varied from −10 dB to 10 dB. It is obvious from Figure 3 that proposed approach is superior to other approaches and attains optimal at all SNRs. At SNR as low as −10 dB, the output SINR of proposed approach is approximately 10.5 dB, which is 6.5 dB higher than the MVDR-Smooth approach 4 dB, and 24.5 dB higher than the EBB approach −14 dB. As the power level of desired signals increases, the output SINR of proposed approach and MVDR-Smooth approach all increases linearly. The MVDR-Smooth approach is approximately 6.5 dB lower than the proposed approach at all considered SNRs. Moreover, It shows that EBB approach is insensitive to the SNR level. The EBB approach stays around −14 dB at all SNRs.

### 4.4. Simulation Example 4

**Figure 4:**
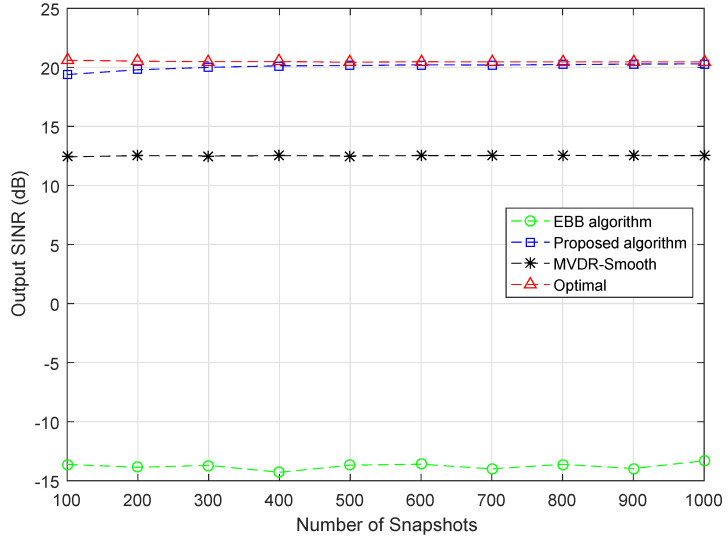
Output SINR versus the number of snapshots, INR (Group 1) = 5 dB, INR (Group 2) = 25 dB, SNR = 0 dB.

In the fourth example, to further validate the performance of the proposed method, the convergence ability of the proposed approach is tested. As illustrated in the figure, Figure 4 demonstrates the output SINR of different beamformers versus the number of snapshots for the fixed SNR and INR. The INR are set as 5 dB and 25 dB for interference Group 1 and Group 2, respectively. The SNR is fixed at 0 dB. With other conditions unchanged, the SINR curves are drawn against the number of snapshots varying from 100 to 1000. It can be clearly observed that the proposed approach enjoys the best performance among the compared beamformers and is able to reach nearly the optimal performance. In general, the proposed approach is not sensitive to the number of snapshots.

### 4.5. Simulation Example 5

**Figure 5:**
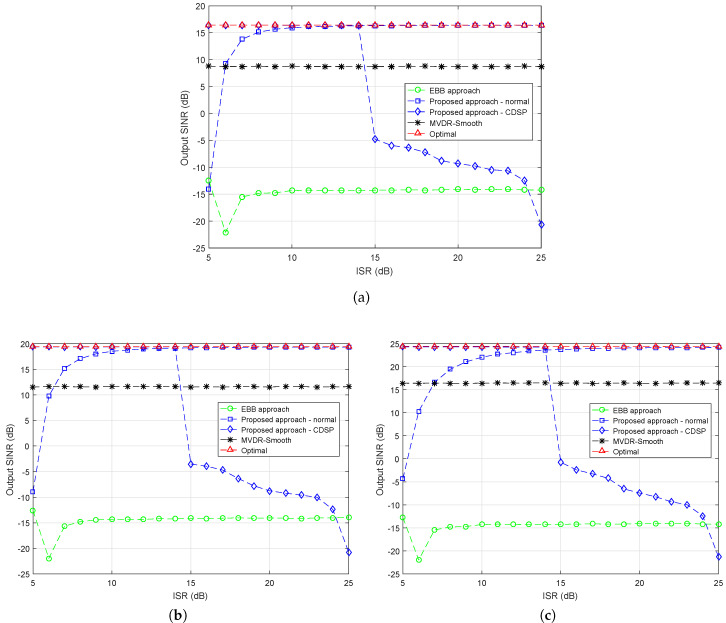
Output SINR versus ISR (Group 3), INR (Group 1) = 5 dB, INR (Group 2) = 25 dB, (**a**) SNR = −5 dB, (**b**) SNR = 0 dB, (**c**) SNR = 5 dB.

In the four examples concerned above, there exists an assumption that only two groups of interferences are employed, one for the normal interferences and one for the CDSP interferences. In this example, we further extend the scenario by adding one additional group of interferences $I_{7},I_{8},I_{9}$ with DOAs $\left\{-10^{\circ },0^{\circ },10^{\circ }\right\}$ named by Group 3. The DOAs of Group 1 and 2 are set the same as before, the ISR of Group 1 and Group 2 are fixed at 5 dB and 25 dB. The output SINR curves of different approaches are displayed versus the ISR of Group 3 varying from 5 dB to 25 dB. We plot the output SINR curves at SNR of −5 dB in Figure 5a, 0 dB in Figure 5b and 5 dB in Figure 5c. Since the ISR of Group 3 varies and it is essential to determine its division according to the power extent. Therefore, in Figure 5a–c, except the curves of compared approaches, two curves of proposed approach denote to for different division plans are drawn respectively, i.e., the blue curve marked with squares stands for the output SINR when Group 3 is processed as normal interferences, and the blue curve marked with diamonds is for Group 3 be divided into CDSP interferences. In all three figures, proposed approach achieves the optimal at approximately 10 dB higher than the desired signals, which means that it is suitable to consider the interferences as normal interferences when ISR >10 dB. It is worth noting that this result is consistent with Example 1. On the other hand, it is shown that at ISRs from 5 dB to 10 dB, dividing the Group 3 into the CDSP interferences obtains better performance. In terms of other approaches, the performance of MVDR-Smooth is steady as expected, but its output SINR gets higher as SNR gets larger, it is around 8 dB in Figure 5a, 12dB in Figure 5b and 17dB in Figure 5c at all ISR levels.

## 5. Conclusions

A new blind beamforming approach for coherent signals is proposed in this paper. When the prior information about desired signals is unavailable, the proposed approach can effectively suppress the interferences and noise, and extract original signal sequence from the received data. The proposed approach is mainly based on the proposed doubly covariance matrix reconstruction concept. The covariance matrices are reconstructed respectively with two methods. Based on the reconstructed INCM, the unblinding procedure is implemented to obtain the CSV of desired coherent signals. Simulation examined the output SINR improvement of proposed approach compared to other approaches. The proposed approach avoids the freedom degree loss since no smooth procedure is involved. It has been verified through simulations that our proposed approach attains optimal when the ISR of normal interferences are around 10 dB, and this ISR can be easily met in many scenarios. In Example 5, we further explored a more complicated scenario, by adding an additional interference group. The simulation results illustrate that when ISR is stronger than 10 dB, the interferences can be considered and processed as normal interferences, and at ISR <10 dB, dividing the interferences into CDSP is more appropriate, which is consistent with the result in Example 1. It should be noted that the proposed approach is applicable for the linear array despite of the way the elements are spacing, i.e., the proposed approach can be used in both the equispaced and non-equispaced array since the only difference is the steering vector. In our future work, the experiments based on real antenna array will be taken into consideration to further validate our approach in practice.
